# Interactions Between Natural Products and Tamoxifen in Breast Cancer: A Comprehensive Literature Review

**DOI:** 10.3389/fphar.2022.847113

**Published:** 2022-06-02

**Authors:** Christine Yen, Fan Zhao, Zhichao Yu, Xiaoshu Zhu, Chun Guang Li

**Affiliations:** ^1^ Chinese Medicine Centre, Western Sydney University, Sydney, NSW, Australia; ^2^ School of Health Sciences, Western Sydney University, Sydney, NSW, Australia; ^3^ College of Chinese Medicine, College of Integrated Chinese and Western Medicine, Nanjing University of Chinese Medicine, Nanjing, China; ^4^ College of the First Clinical Medical, Nanjing University of Chinese Medicine, Nanjing, China; ^5^ NICM Health Research Institute, Western Sydney University, Westmead, NSW, Australia

**Keywords:** breast cancer, natural products, herbal medicines, tamoxifen, pharmacodynamic, pharmacokinetic, interactions, mechanisms

## Abstract

**Introduction:** Tamoxifen (TAM) is the most commonly used hormone therapeutic drug for the treatment of estrogen receptor-positive (ER+) breast cancer. 30%–70% of clinical breast cancer patients use natural products, which may increase the likelihood of drug interactions.

**Objective:** To evaluate the evidence for the interactions between natural products and TAM in breast cancer.

**Methods:** Electronic databases, including PubMed, CINAHL Plus (*via* EbscoHost), European PMC, Medline, and Google Scholar, were searched for relevant publications. The search terms include complementary and alternative medicine, natural products, plant products, herbs, interactions, tamoxifen, breast cancer, and their combinations.

**Results:** Various *in vitro* and *in vivo* studies demonstrated that the combined use of natural products with TAM produced synergistic anti-cancer effects, including improved inhibition of tumor cell growth and TAM sensitivity and reduced side effects or toxicity of TAM. In contrast, some natural products, including *Angelica sinensis* (Oliv.) Diels [Apiaceae]*, Paeonia lactiflora* Pall*.*, *Rehmannia glutinosa* (Gaertn.) DC.*, Astragalus mongholicus* Bunge, and *Glycyrrhiza glabra* L. [Fabaceae], showed estrogen-like activity, which may reduce the anti-cancer effect of TAM. Some natural products, including morin, silybin, epigallocatechin gallate (EGCG), myricetin, baicalein, curcumin, kaempferol, or quercetin, were found to increase the bioavailability of TAM and its metabolites *in vivo*. However, three are limited clinical studies on the combination of natural products and TAM.

**Conclusion:** There is evidence for potential interactions of various natural products with TAM in pre-clinical studies, although the relevant clinical evidence is still lacking. Further studies are warranted to evaluate the potential interactions of natural products with TAM in clinical settings.

## 1 Introduction

Breast cancer is one of the more frequently diagnosed cancers and the leading cause of cancer deaths in women worldwide (Jemal et al., 2011). The latest cancer statistics in 2019 show that breast cancer accounts for around 30% of all the new cancer diagnoses in women in the United States, of which approximately 1 in 6.5 (15.5%) will die of cancer (Siegel et al., 2019). According to the presence of immunohistochemistry markers, such as estrogen receptor (ER), progesterone receptor (PR), or human epidermal growth factor 2 (HER2) molecular markers, breast cancer can be classified into three main subtypes: hormone receptor-positive (ER+, PR+, HER2−), HER2 positive (ER−, PR−, HER2+), and triple-negative breast cancer TNBC (ER−, PR−, HER2−). Among them, estrogen receptor-positive (ER+) breast cancer accounts for approximately 60%–75% of all breast cancer patients. Adjuvant endocrine therapy is an established effective systemic treatment for most ER+ breast cancers and has become the most widely employed therapy to treat hormone receptor-positive breast cancers.

Tamoxifen (TAM) is a selective estrogen receptor modulator, acting as an estrogen antagonist in the breast while acting as an estrogen agonist in the uterus. TAM binds to the estrogen receptor to form a nuclear complex that blocks the estrogen receptor transcriptional activity, thereby reducing DNA synthesis and inhibiting the effect of estrogen (Ali et al., 2016). TAM demonstrated clinically significant effects of reducing breast cancer recurrence rate by 40%–50% and could reduce the mortality and temporary remission rate after treatment and may reduce mortality and increase remission rate (Karn et al., 2010). However, on top of the concern for survival and growth of TAM-tolerant cells, there are adverse events (AE) associated with TAM therapy, including hot flashes, increased risk of endometrial cancer, and, in rare instances, liver abnormalities (Viedma-Rodríguez et al., 2014).

Recently there has been an increase in studies on the anti-cancer properties of natural products (Wu et al., 2017; Lichota and Gwozdzinski, 2018) and increasing use of various natural products by cancer patients (Chen XW et al., 2011). Of all the cancer patients, breast cancer patients, in particular, are more likely to turn to complementary and alternative medicine (CAM) treatment (Wanchai et al., 2010). An Australian study found that 55% of patients using biological-based CAM and 80% of patients using nonbiological-based CAM did not discuss their use of CAM with their oncologist (Kremser et al., 2008). Many studies report that herbal or dietary supplements can affect various molecular targets and signaling pathways, leading to the possible use of breast cancer combination therapy (Chen et al., 2011a; Mansour et al., 2012; Oleaga et al., 2012; Pawlik et al., 2016). However, there is still a lack of evidence-based information or guidance for clinicians and consumers on the interaction between herbs and drugs used to treat and prevent cancer (Sparreboom et al., 2004).

This review aimed to evaluate the evidence available from the literature on the combined use of natural or plant products and TAM in breast cancer studies and identify the current gaps and possible future studies for improving treatment strategies for breast cancer.

## 2 Literature Search

Electronic databases, including PubMed, CINAHL Plus (*via* EbscoHost), European PMC, Medline, and Google Scholar, were searched for relevant publications. The search terms include “complementary and alternative medicine,” “natural product*,” “plant product*,” “herb*,” “interaction*,” “tamoxifen,” “breast cancer,” and “endoxifen” and their combinations. The search results include publications from their inceptions to 2021 on the single herbs or a combination of herbs or natural products except for unpublished studies or abstracts that do not have sufficient data for analysis. The exclusion criteria are as follows: articles that did not analyze herb and TAM in combination or separately or comment on herb-TAM interactions; articles that included a cocktail of endocrine agents; non-English publications (not including articles with English abstracts, tables, or figures); and contribution articles such as letters, editorials, commentaries, or letters to the editor.

## 3 Results

A total of 100 relevant articles were identified among 1,518 entries found in the literature search, including 24 pharmacokinetic studies on herbal medicines and 76 on pharmacodynamics studies. A summary of the findings is shown in Tables 1–6, with Table 1 on pharmacokinetics interactions, Table 2, 3 on synergetic anti-effects, Table 4 on sensitization, Table 5 on side effects or toxicity of combined use, and Table 6 on antagonizing effects.

**TABLE 1:** Reported pharmacokinetics interactions of natural products and tamoxifen.

Herb	Evidence	Dosage of tamoxifen	Dosage of herb	AUC^a^ or C_max_ ^b^ of tamoxifen	AUC or C_max_ of 4-OHT	CL/F^c^	AB (%)^d^	MR (%)^e^	Suggested mechanism
Morin	Animal study Male Sprague–Dawley rats, 270–300 g (Shin et al., 2008)	10 mg/kg o.p.	3 or 10 mg/kg o.p.	Increase	Increase	Decrease	Increase	Decrease	-
Silibinin/silybin	Animal study Male Sprague–Dawley rats, 270–300 g (Kim et al., 2010)	10 mg/kg o.p.	0.5, 2.5, or 10 mg/kg o.p.	Increase	Increase	-	Increase	-	Inhibit CYP3A4 and 2C9
EGCG^f^	Animal study Male Sprague–Dawley rats, 270–300 g (Shin & Choi, 2009)	10 mg/kg o.p.	0.5, 3, or 10 mg/kg o.p.	Increase	Increase	-	Increase	Decrease	-
Myricetin	Animal study Male Sprague–Dawley rats, 270–300 g (Li et al., 2011b)	10 mg/kg o.p.	0.4, 2, or 8 mg/kg o.p.	Increase	Increase	-	Increase	Decrease	Inhibit CYP3A4 and 2C9
Baicalein	Animal study Male Sprague–Dawley rats, 270–300 g (Li et al., 2011a)	10 mg/kg o.p.	0.5, 3, or 10 mg/kg o.p.	Increase	Increase	Decrease	Increase	Decrease	Inhibit CYP3A4
Curcumin	Animal study Male Sprague–Dawley rats, 270–300 g (Cho et al., 2012)	10 mg/kg o.p.	0.5, 2.5, or 10 mg/kg	Increase	Increase	-	Increase	Decrease	-
Kaempferol	Animal study Male Sprague–Dawley rats, 270–300 g (Piao et al., 2008)	10 mg/kg o.p.	2.5 or 10 mg/kg o.p.	Increase	None	-	-	None	-
Quercetin	Animal study Female Sprague–Dawley rats, 270–300 g (Shin et al., 2006)	10 mg/kg o.p.	2.5, 7.5, or 15 mg/kg o.p.	Increase	Increase	-	Increase	Decrease	-

a AUC: area under the plasma concentration-time curve.

b C_max_: peak plasma concentration.

c CL/F: systemic clearance.

d AB (%): absolute bioavailability.

e MR (%): metabolite-parent ratio.

f ECGC: epigallocatechin gallate.

**TABLE 2:** Reported synergetic effects of a combination of inhibiting tumor growth, inhibiting proliferation, or inducing apoptosis.

Herb/formulations	Evidence	Dosage of tamoxifen	Dosage of herb	Effects	Suggested mechanism
Genistein	BT-474 cell lines (Mai et al., 2007)	5 μM	25 μM	Inhibit cell growth and apoptosis	Downregulate survivin, HER1, HER2, HER3, and ER-α expression
Z-Ligustilide	MDA-MB-231 cell lines (Ma et al., 2017)	5 μM	50 μM	Induce apoptosis and S and G2/M phases cell cycle arrest	Downregulate cyclin A, cyclin E, CDK1, and CDK2 levels, upregulate p21, p27, MTA1 levels, reactive the ERα expression, and transcriptional activity
SCS	MCF-7, MDA-MB-231 cell lines (Yaacob et al., 2014)	2.5, 5 μM	8.5 or 10.0 μg/ml	Inhibit proliferation, increase apoptosis	Modulate mitochondrial membrane and active caspase-8 and caspase-9
GTE	MCF-7, ZR75, and T47D cell lines (Sartippour et al., 2006)	5–10 μg/ml	0.2–40 μg/ml	Inhibit proliferation, increase apoptosis	Decrease level of ER-α, inhibit the stimulation of p44/42 MAPK activity
*Viscum album* L.	MCF-7 cell line (Weissenstein et al., 2019)	1 μM endoxifen	10 μg/ml	Inhibit proliferation, increase apoptosis, and induce cell cycle arrest	-
FE	MDA-MB-231 and MCF-7 cell lines (Zhang et al., 2013)	10 μM	200 μg/ml	Enhance cytotoxicity, Synergistic Induce apoptosis and cell cycle arrest	Downregulate the expression of BCL-xl and Mcl-1, upregulate the expression of BAX, modulate ERK and AKT phosphorylation, increase ROS level, and reduce GSH level
*Trifolium pratense*	MCF-7 and MDA-MB-231 cell lines (Vlaisavljevic et al., 2014)	1.55–24.88 μM, MCF-7 cell; 11–176 μM, MDA-231 cell	142–2272 μg/ml, MCF-7 cell; 210.5–3368 μg/ml, MDA-231 cell	Increase cytotoxic effect	-
*BreastDefend*	MCF-7 cell lines (Cheng et al., 2017)	1 μM 4-OHT	10 μg/ml	Inhibit proliferation and induce apoptosis	Upregulate c-PARP, BRAF, p21mRNA levels, downregulate FN1 Bcl-2 mRNA levels
Huaier	BALB/c nu/nu female mice (Qi et al., 2016)	5 mg/kg i.g.	0.5 mg/μl i.g.	Reduce tumorigenesis and metastasis	Upregulate Bax, Beclin-1, Lc3b, and downregulate Bcl-2, Cyclin D1 level
GTE	Ovariectomized nude mice (Sartippour et al., 2006)	20 mg pellet, subcutaneously inoculated	2.5 g/l in drinking water	Inhibit tumor growth, suppress angiogenesis, increase larger areas of necrosis and lower tumor blood vessel density	Decrease level of ER-α
	C3H/OuJ mice (Sakata et al., 2011)	10 mg pellet, subcutaneously inoculated	0.1 or 1% in drinking water	Decrease the tumor incidence and AgNOR counts	-
*BreastDefend*	Nu/Nu immune-compromised female ovariectomized mice (Cheng et al., 2017)	5 mg/pellet, subcutaneously inoculated	100 mg/kg i.g.	Inhibit tumor growth, enhance apoptosis	Increase apoptotic bodies, upregulate expressions of BRAF and p21, downregulate pro-apoptotic Bcl-2 protein

**TABLE 3:** Reported synergetic effects of inhibiting proliferation or inhibiting tumor growth

Herb formulations	Evidence	Dosage of tamoxifen	Dosage of herb	Effects	Suggested mechanism
Tangeretin	MCF-7/6 cell lines (Bracke et al., 1999)	1 µM	0.1 mM	Inhibit proliferation	-
Genistein	MCF-7, MDA-231, MDA-435 cell lines (Tanos et al., 2002)	0.1, 1 µM	1.0–10 mg/ml	Inhibit proliferation	-
Canola oil	MCF-7 and T47D cell lines (Cho et al., 2010)	10 μM	1 mM	Inhibit proliferation	-
EGCG	MCF-7 cell lines (Sakata et al., 2011)	0.001–1 µM	0.01–10 µM	Inhibit proliferation	-
HP03	MCF-7 cell lines (Chang et al., 2018)	6.4 μM	10.30,100 μg/ml	Inhibit proliferation	Downregulate Psen2, PGR, CTSD mRNA expression
SHD	MCF-7 cell lines (Xu et al., 2016)	3 μg/ml	2 mg/ml	Inhibit cell growth and induct apoptosis	Downregulate Aurora A
GbE	SD rats injected with MNU (Dias et al., 2013)	10 mg/kg i.g.	100 mg/kg i.p.	Reduce tumor area and increase tumor necrosis area	-
SDG and FO	Ovariectomized BALB/c, nu/nu athymic mice (Saggar et al., 2010)	5 mg pellet, subcutaneously inoculated	FO (38.5 g/kg diet), SDG (1 g/kg in diet)	Inhibit tumor growth	Downregulate estrogen-sensitive gene CD1, PGR, ER-a, ER-b, PS2 mRNA expression levels, growth factor signaling gene EGFR, IGF-1R, BCL2, VEGF, HER2 mRNA expression levels, PMAPK, PAKT, AIBI, PHER2 protein expression levels
Hes, Pip, and BV	MCF-7 and T47D cell lines (Khamis et al., 2018)	-	-	Increase apoptosis, arrest cell cycle in G2/M, and G0/G1 phase	Upregulate Bax mRNA level, downregulate Bcl2 mRNA level, decrease the two breast cancer-related receptors, EGFR and ERα mRNA
Persin	MCF-7 and T47D cell lines (Roberts et al., 2007)	10 nmol/L 4-OHT	2.76 μmol/L	Increases apoptosis	Downregulate ERα mRNA level, upregulate Bim mRNA level
Huaier	MCF-7 and T47D cell lines (Qi et al., 2016)	10 μM	4 mg/ml	Induce autophagy and apoptosis	Suppress the AKT/mTOR pathway

**TABLE 4:** Reported evidence of increasing sensitization.

Herb	Evidence	Dosage of tamoxifen	Dosage of herb	Effects	Suggested mechanism
Z-Ligustilide	MCF-7 TR5 cell lines (Qi et al., 2017)	2.5 μM	50 μM	Induce autophagy and apoptosis	Restore autophagy-degrades Nur77
Curcumin	MCF-7/LCC2 cell lines (Jiang et al., 2013)	2.5 μM	1.78 μM	Increase sensitivity to 4-OHT	-
	MCF-7/LCC9 cell lines (Jiang et al., 2013)		4.09 μM		
Antrodia cinnamomea extract	MCF-7/Tam-R (Lin et al., 2018)	10 μM	100, 150, 200 ug/ml	Inhibit cell proliferation	-
Evn-50	MCF-7/Tam-R (Hu et al., 2012)	5 μmol/L	50 μg/ml	Inhibit cell viability, induce apoptosis and G2 phases cell cycle arrest	Downregulate AKT and MAPK pathway
Persin	MCF-7/Tam-R (McCloy et al., 2013)	7.5 μM	1 μg/ml	Reverses 4-OHT resistance, induce apoptosis	Mediate ERS signal
JEKHT^a^	MCF-7/LCC9 cell lines (De Oliveira Andrade et al., 2019)	125 nM	1 mg/ml	Inhibit cell proliferation	-

a JEKHT: composed of *Paeonia* (*Paeonia lactiflora* Pall.), Korean angelica root (*Angelica gigas* Nakai), Asparagi Tuber (*Asparagus cochinchinensis* (Lour.) Merr.), White atractylis (*Atractylodes lancea* (Thunb.) DC.), *R. glutinosa* (*Rehmannia glutinosa* (Gaertn.) DC.), Dried orange peel (*Citrus aurantium* L.), Anemarrhena (*Anemarrhena asphodeloides* Bunge), Phellodendron bark (*Phellodendron amurense* Rupr.), Licorice (*Glycyrrhiza glabra* L.), Ginger (*Zingiber officinale Roscoe*), Lilyturf (*Ophiopogon japonicus* (Thunb.) Ker Gawl.), Jujube (*Ziziphus jujuba Mill*.**)**

**TABLE 5:** Reported evidence of reducing toxic and side effects of tamoxifen.

Adverse effects of TAM	Herb	Evidence	Dosage of tamoxifen	Dosage of herb	Effects
Hot flushes	Cimicifuga Racemose (BNO 1055)	Clinical research (intervention group, *n* = 90) (Hernández Muñoz & Pluchino, 2003)	20 mg per day orally	20 mg herbal drug	Reduce the number and severity of hot flushes
		Prospective observational study (*n* = 50) (Rostock et al., 2011)		20–80 mg herbal drug	Reduce the total MRS II score and improve hot flashes, sweating, sleep problems, and anxiety
Endometrial lesions	Dang-Qui	Population-based study (intervention group, *n* = 77)Wu et al. (2014)	-	-	Reduce the hazard ratio for the development of endometrial cancer
	CHPs containing coumestrol, genistein, and/or daidzein	Population-based study (intervention group, *n* = 9,652) (Hu et al., 2015)			Negatively correlate with subsequent endometrial cancer risk
	CHPs	Population-based study (intervention group, *n* = 20,466) (Tsai et al., 2014)			Decrease the risk of subsequent endometrial cancer
	JEKHT^a^	Animal research, SD rats (De Oliveira Andrade et al., 2019)	15 mg/kg	500 mg/kg	Prevented the development of premalignant endometrial lesions
Liver damage	PFDB	Animal research, SD rats (Rahate & Rajasekaran, 2015)	45 mg/kg	100, 200 mg/kg	Reduced glutathione, glutathione peroxidase, superoxide dismutase and catalase, the lipid peroxidation in the liver tissue, and hepatocellular necrosis
	GTE	Animal research, Albino rats (El-Beshbishy, 2005a)	45 mg/kg	Orally administered 1.5% GTE as the sole source of drinking water	Scavenge free radicals and protect against oxidative stress induced by TAM intoxication
	Dried apple enriched with mandarin juice	Animal research, Wistar rats (Codoñer-Franch et al., 2013)	1.54 mg/kg, three times a day	0.745 g/day	Decrease aminotransferases, CGs, and 8OHdG and increase α-tocopherol
	Dimethyl Dimethoxy Biphenyl Dicarboxylate	Animal research, Albino rats (El-Beshbishy, 2005b)	45 mg/kg	200 mg/kg	Increase antioxidant enzymes (glutathione-S-transferase, glutathione peroxidase, and catalase) and reduce glutathione concomitant with significant decrements in TBARS and liver transaminases, sGPT and sGOT levels

a JEKHT: composed *Paeonia* (*Paeonia lactiflora* Pall.), Korean angelica root (*Angelica gigas Nakai*), Asparagi Tuber (*Asparagus cochinchinensis* (Lour.) Merr.), White atractylis (*Atractylodes lancea* (Thunb.) DC.), *R. glutinosa* (*Rehmannia glutinosa* (Gaertn.) DC.), dried orange peel (*Citrus aurantium* L.), *Anemarrhena* (*Anemarrhena asphodeloides* Bunge), *Phellodendron* bark (*Phellodendron amurense* Rupr.), Licorice (*Glycyrrhiza glabra* L.), Ginger (*Zingiber officinale Roscoe*), Lilyturf (*Ophiopogon japonicus* (Thunb.) Ker Gawl.), Jujube (*Ziziphus jujuba Mill*.**)**

**TABLE 6:** Reported evidence of reducing anti-cancer effects.

Herb	Evidence	Dosage of tamoxifen	Dosage of herb	Effects	Suggested mechanism
Xanthorrhizol	BALB/c athymic female nude mice (Noomhorm et al., 2014)	4.6 mg/kg mice/2 days	0.1, 0.2, 0.4 mg/kg	Increased tumor volumes, a larger tumor size	Upregulate protein expression of P38 and P27 (Kip1)
SWT^a^	MCF-7-implanted athymic nude mice (Chen et al., 2013)	2.3 mg/kg	4.255 g/kg	Reversed Tam-induced anti-proliferative effects on tumor weight and tumor volume	Increase ERα and N-cadherin expression, upregulate ERK, AKT, P38, p27 (Kip1) level
BZT^b^	Female nude and male ICR mice (Li et al., 2019)	4 mg/kg	2, 9 g/kg	Attenuate the effectiveness of tamoxifen and reduce the concentrations of endoxifen and 4-OH-tamoxifen in tumor-bearing mice	Inhibit CYP450 enzyme activity in rat liver microsomes
Tangeretin	Nude mice (Bracke et al., 1999)	30 µM in drinking water	0.1 mM in drinking water	Neutralize tamoxifen’s inhibitory effect	-

a SWT: composed of *Angelica sinensis* (Oliv.) Diels [Apiaceae], *Ligusticum chuanxiong* Hort., *Paeonia lactiflora* Pall., and Radix Rehmanniae Preparata (*Rehmannia glutinosa* (Gaertn.) DC.).

b BZT: consisting of *Lilium brownii* var. viridulum Baker (*L. brownii*) and *Anemarrhena asphodeloides* Bunge.

TAM has a relatively lower affinity for the estrogen receptor (Brauch et al., 2009; Kim et al., 2021). After a single oral administration, the maximum plasma concentration of the parent drug and demethylated metabolites can be reached within a few hours. Within the therapeutic margin, it is highly bound to serum proteins, which will then undergo extensive liver metabolism. Metabolism of TAM takes place mainly through two main pathways, 4-hydroxylation and N-demethylation, from which TAM is converted to primary metabolites 4-hydroxy-tamoxifen and N-desmethyl-tamoxifen (Klein et al., 2013).

Both primary metabolites are further converted to secondary metabolite endoxifen, catalyzed by CYP3A4/5 and CYP2D6 (Desta et al., 2004). Endoxifen competes with estrogen and binds to ER with nearly 100 times higher affinity than TAM (Brauch et al., 2009). It exhibits estrogenic activity by binding to ligand-regulated transcription factors ERα/ERβ. The resulting nuclear complex affects the transcription of estrogen-responsive genes, which is responsible for the production of various growth-promoting signals (Riggs and Hartmann, 2003).

### 3.1 Pharmacokinetic Interactions

Several *in vitro* and *in vivo* studies on the bioavailability and metabolism of TAM and its metabolite 4-hydroxy-tamoxifen found that morin (Shin et al., 2008), silybin (Kim et al., 2010), Epigallocatechin gallate (EGCG) (Shin and hoi, 2009), myricetin (Li et al., 2011b), baicalein (Li et al., 2011a), curcumin (Cho et al., 2012), kaempferol (Piao et al., 2008), and quercetin (Shin et al., 2006) significantly changed the pharmacokinetics of oral TAM, resulting in reduced systemic clearance (CL/F); increased the area under the plasma concentration-time curve (AUC_0-∞)_, in the peak plasma concentration (Cmax); and increased absolute and relative bioavailability, which may be the result of reducing first-pass metabolism in the intestine and liver.

Among these, morin and kaempferol had no significant effect on the formation of 4-hydroxy-tamoxifen (Piao et al., 2008; Shin et al., 2008). However, silybin, EGCG, myricetin, baicalein, curcumin, and quercetin significantly changed AUC_0-∞_ and the metabolite-maternal ratio (MR) of 4- hydroxy-tamoxifen, indicating that it can significantly increase the bioavailability of TAM and affect the formation of 4-hydroxy-tamoxifen (Shin et al., 2006; Shin and Choi, 2009; Kim et al., 2010; Li et al., 2011a; Li et al., 2011b; Cho et al., 2012). These findings highlight that natural or plant products can interfere with the pharmacokinetics of TAM, as seen in Table 1. If clinical studies further confirm the results of the above pharmacokinetic studies, an adjustment of clinical TAM doses may be needed when co-administrated with these products to avoid potential drug–herb interactions.

On the contrary, studies found no interactions between certain natural products and TAM. For example, *Ginkgo biloba* L. extract is an effective herb widely used in diseases including breast cancer (Jacobs and Browner, 2000). A prospective open-label cross-over on the effect of clinical *Ginkgo biloba* L. extract co-administration on the pharmacokinetics of TAM found that there was no significant difference in drug concentration and toxicity before and after 3-week *Ginkgo biloba* L. extract treatment according to the LC-MS/MS results of 20 women with early breast cancer who took TAM received *Ginkgo biloba* L. extract (EGb 761) (120 mg twice daily), indicating that co-administration of *Ginkgo biloba* L. extract would not significantly affect the pharmacokinetics of TAM (Vardy et al., 2013).

Fucoidan is a polysaccharide compound naturally occurring in brown algae. *In vivo* and *in vitro* studies have shown that fucoidan can prevent and treat breast cancer, promyelocytic leukemia, colon cancer, breast cancer, liver cancer, and melanoma (Atashrazm et al., 2015). An open-label non-crossover study of patients with active malignant tumors taking letrozole or TAM (n = 10 per group) found that the results of HPLC-CAD (high-performance liquid chromatography with aerosol detector) of patients who took oral fucoidan in the form of fucoidan extract for 3 weeks (500 mg twice daily) showed no significant difference in letrozole, TAM concentration, and the steady-state plasma concentration of TAM metabolites and no adverse effects of fucoidan. These results indicate that, at research, fucoidan does not interact with letrozole and TAM (Tocaciu et al., 2018).

Another study investigating the effects of combined treatment of MCF-7 with a fermented mistletoe preparation (VAE) and TAM reported that the combination of clinically relevant doses of VAE with TAM metabolite endoxifen did not show significant inhibition of TAM metabolism catalyzed by CYP3A4/5 and CYP2D6, indicating mistletoe (*Viscum album* L.) preparations can be combined with TAM without the risk of HDI *in vitro* (Weissenstein et al., 2019).

### 3.2 Pharmacodynamic Interactions

The key mechanisms of action of anti-cancer drugs involve apoptosis, autophagy, and cell cycle arrest (Chen and Karantza-Wadsworth, 2009; Wang et el., 2011; Kon et al., 2013). In the normal state, apoptosis is a highly specific and carefully controlled programmed cell death. Under pathological conditions, especially in cancer, cells lose their ability to induce apoptosis and proliferate uncontrollably. In the catalytic activation of the apoptotic cascade, caspase family proteases and BCL-2 family proteins such as Bcl-2-associated X (BAX) and B-cell lymphoma 2 (Bcl-2) play an important role (Pistritto et al., 2016).

Programmed cell death (PCD) is not limited to apoptosis (type I) PCD but also autophagic (type II) PCD (Bursch et al., 2000). Regulating autophagy can also improve anti-cancer treatment (Moretti et al., 2007). It is an important process in programmed cell death and the phagocytosis and degradation of non-essential or abnormal organelles and proteins (Denton et al., 2012). In the process of autophagy, there is an increase in the expression of autophagy genes Beclin 1, PI3KC3, and LC3-II (Kang et al., 2010; Liu et al., 2010), while p62 can be degraded by autophagy (Bjørkøy et al., 2005). As deregulation of the cell cycle is one of the main characteristics of cancer cells, cell cycle arrest may be an effective strategy to control the abnormal proliferation of cancer cells (Novák and Tyson, 2003).

Genome replication and cell division are regulated by cell cycle progression (Malumbres and Barbacid, 2009). The control and coordination of cell cycle progression involve cyclin-dependent kinases (CDK) and cyclins, including p53, p21, p16, and cdc25 (Malumbres and Barbacid, 2009). Current evidence, summarized in Tables 2, 3, reports that natural or plant products and TAM can target the physiological characteristics of cancer cells, inhibit cell proliferation, and exert anti-cancer effects *via* participation in the aforementioned cellular mechanisms such as apoptosis, autophagy, and cell cycle. There is also evidence of the combined use of some natural or plant products combined with TAM, in which the natural or plant products can significantly increase the sensitivity of drug-resistant cells to TAM *in vitro* (Table 4).

#### 3.2.1 Inhibiting Tumour Cell Growth and Increasing Apoptosis

##### 3.2.1.1 In Vitro Studies

The plant flavonoid hesperidin (HES) is a natural product with obvious anti-tumor effects and is widely present in citrus fruits, piperine (PIP), and bee venom (BV) extracted from black pepper (*Piper nigrum* L.) and long pepper (*Piper longum* L.) (Park et al., 2011; Palit et al., 2015; Deng et al., 2016). A recent study has shown that the combined use of HES, PIP, and BV with TAM showed an enhanced anti-proliferative effect on MCF7 and T47D and can significantly increase cell apoptosis (Khamis et al., 2018). Furthermore, this study reports that the mRNA level of the pro-apoptotic factor BAX is upregulated, whereas the mRNA level of the anti-apoptotic protein Bcl-2 is downregulated (Khamis et al., 2018). It can reduce the expression of breast cancer-related receptors EGFR and ERα and induce the G2/M phase arrest (Khamis et al., 2018). Tangerine, a flavonoid present in oranges, inhibits the growth of MCF-7/6 breast cancer cells in a manner similar to TAM *in vitro*, activates cell adhesion, and prevents invasion (Bracke et al., 1999). The combined use of tangerine with TAM exhibited a synergistic inhibitory effect on the growth of MCF-7/6 cells (Bracke et al., 1999).

Isoflavone genistein is one of the most important phytoestrogens. When combined with TAM, it can further inhibit the proliferation of MCF-7, MDA-231, and MDA-435 cells (Tanos et al., 2002). Another study showed that the combination of genistein and TAM could further inhibit the proliferation of BT-474 cells, increase apoptosis, arrest the cell cycle in the G1 phase, and be involved in the synergistic downregulation of the expression of survivin (Mai et al., 2007). Persin is a novel phytotoxin, which, combined with 4-hydroxytamoxifen on MCF-7 and T-47D cells, increases the sensitivity of cells to TAM, increases cell apoptosis, reduces ERαmRNA expression, and increases B-cell lymphoma 2 interacting mediator of cell death (BIM) mRNA expression (Roberts et al., 2007). Canola oil (CAN) has a low content of saturated fatty acids and a high content of unsaturated fatty acids. When combined with TAM, it exhibits a significant synergistic inhibitory effect on MCF-7 and T-47D cell growth (Cho et al., 2010).

Z-Ligustilide, a phthalate compound, is the primary active ingredient of the Chinese herbal medicine *Angelica sinensis* (Oliv.) Diels. When used in combination with TAM, it has been found to increase the growth inhibitory effect in ERα-breast cancer cells MDA-MB-231 and induce cell apoptosis and cell cycle arrest in S and G2/M phases (Ma et al., 2017). A combination of Z-ligustilide with TAM is also found to activate the expression and transcriptional activity of ERα of MDA-MB-231 cells and reduce the expression of metastasis-associated protein 1 (MTA1) (Yaacob et al., 2015).

When an active subcomponent (SCS) in the leaves of the traditional medicinal plant flowering shrub (*Strobilanthes crispa* (L.) Blume) in Malaysia and Indonesia used in combination with TAM under low doses of antiestrogens, it has been reported to synergistically induce cell cycle arrest in MCF-7 and MDA-MB-23 in the G1 phase, *via* mechanisms involving the regulation of cyclin D1, p21 CDK inhibitor and p53 tumor suppressor protein, modulation of mitochondrial membrane potential, and activation of caspase-8 and caspase-9 (Yaacob et al., 2014). The study also reports that the anti-cancer effect of SRS involves the downregulation of ERα protein; however, it is independent of ER-mediated mechanism in MDA-MB-231 cells (Yaacob et al., 2014).

In addition to water, green tea (*Camellia sinensis* (L.) Kuntze [Theaceae]) is the most widely consumed beverage in the world; current studies have reported that drinking green tea can improve the prognosis of breast cancer (Nagata et al., 1998; Fujiki et al., 1999). The combination of green tea extract (GTE) and TAM can synergistically inhibit the proliferation of MCF-7 cells, with the underlying mechanism relating to the inhibition of MAPK signal transduction (Sartippour et al., 2006). The combination of the main active ingredient of green tea epigallocatechin-3-gallate (EGCG) and TAM also exhibited significant inhibition of MCF-7 cell proliferation (Sakata et al., 2011).

Mistletoe (*Viscum album* L.) derived medicinal preparations are widely used and registered as medicines in many European countries. Clinical studies have reported significant anti-cancer effects of *Viscum album* L. extracts (VAE) in breast cancer patients (Kienle et al., 2009; Tröger et al., 2014). A clinical study on the combined use of mistletoe with the active metabolite of TAM (E/Z) endoxifen reports that clinically relevant doses of VAE did not affect the growth inhibition and cytotoxicity effect of (E/Z) endoxifen on tumor cells and could significantly induce cell cycle in the G0/G1 phase (Weissenstein et al., 2019).

Fucoidan extract (FE) in combination with TAM has been reported with significant induction of cell growth inhibition, apoptosis, and cell cycle modification in MDA-MB-231 and MCF-7 cells (Zhang et al., 2013). The study also found significant downregulation in expression levels of anti-apoptotic proteins Bcl-xL and Mcl-1; significant reduction in the phosphorylation of ERK and Akt in MDA-MB-231 cells but an increased ERK phosphorylation in MCF-7 cells; additionally, a synergistic increase in intracellular ROS level of breast cancer cells and reduction in the level of intracellular glutathione (Zhang et al., 2013).

Huaier, also known as *Trametes robiniophila* Murr., has been used as a traditional Chinese medicine in China for about 1,600 years, with its extracts documented for anti-tumor effects, including inhibition of cell proliferation, anti-metastasis, interference with tumor angiogenesis, induction of autophagic cell death, and tumor-specific immune regulation (Pan et al., 2019). In MCF-7 and T47D cells, the Huaier extract combined with TAM can reduce cell viability and synergistically induce cell autophagy, apoptosis, and G0/G1 cell cycle arrest *via* the inhibition of the AKT/mTOR signaling pathway (Qi et al., 2016).

Red clover, *Trifolium pratense* L., is a member of the legume family, used as a health food for humans, and has been extracted for various isoflavone preparations used in nutritional supplements (Vlaisavljevic et al., 2014). The combination of hydroethanolic extract of red clover and TAM can significantly reduce the survival of MCF-7 and MDA-MB-231 cells. The CI values across different dose combinations on the two cell lines were less than 1, implying that all combinations exhibited a synergistic effect (Khazaei and Pazhouhi, 2019). At the same time, the DRI value of TAM > 1 indicates that the TAM dose can be reduced under a given therapeutic effect (Khazaei and Pazhouhi, 2019).

HPC 03 herbal formula contains extracts of *Angelica gigas* Nakai, *Ligusticum officinale* (Makino) Kitag, and *Cinnamomum cassia* (L.) J. Presl*.* Combined with TAM, they can inhibit the proliferation of MCF-7 cells and reduce the mRNA expression of estrogen-responsive genes: Psen2, PGR, and CTSD (Chang et al., 2018). BreastDefend^®^ (BD) is a dietary supplement formula that includes *Ganoderma lucidum* (Leyss ex Fr.) Karst*.*, *Curcuma longa* L., and quercetin. Combined with 4-hydroxytamoxifen (4-OHT), it exhibits significant inhibition of cell proliferation and induction of MCF-7 cell apoptosis (Cheng et al., 2017). The study evaluated the effect of 4-OHT and BD combinations on gene expression involved with apoptosis and TAM resistance. At the translation level, increased expression levels of BRAF and p21 mRNA and suppressed expression levels of FN1 and Bcl-2 in MCF-7 cells were detected (Cheng et al., 2017). Sanhuang Decoction (SHD) comprises *Astragalus mongholicus* Bunge, *Rheum palmatum* L. [Polygonaceae], and *Curcuma longa* L. in a ratio of 3:1:1 (Xu et al., 2016). The combination of SHD and TAM exhibited additive effects on MCF-7 cell growth inhibition and apoptosis induction (Xu et al., 2016). It can also inhibit the growth of breast cancer cells by downregulating the Aurora A expression and enhancing the chemosensitivity to other anti-tumor drugs (Xu et al., 2016).

##### 3.2.1.2 In Vivo Studies

Ginkgo leaf extract (GbE) is an effective herbal medicine widely used as a CAM for diseases including breast cancer (Jacobs and Browner, 2000). In a study adopting 7,12-dimethyl-benz(a)anthracene- (DMBA-) induced breast cancer animal model, the combined use of GbE and TAM was demonstrated to a significant reduction in the tumor area, increase in the tumor necrosis area, and reduction in the number of proliferating cell nuclear antigen- (PCNA-) positive cells (Dias et al., 2013). The combined use of Huaier extract and TAM *in vivo* can enhance the inhibitory effects of TAM on the growth of subcutaneous tumors of MCF-7 breast cancer cells and synergistically induce autophagy and apoptosis in ER+ breast cancer cells *via* suppression of the AKT/mTOR pathway (Qi et al., 2016).

In a study involving an MCF-7 cell xenograft mouse model, it was reported that the combination of green tea extract (GTE) and TAM significantly inhibit the growth and angiogenesis of tumor tissues (Sartippour et al., 2006). The study also reports that the combination of significant reduction expression levels of ER-α, ∼50% inhibition of transcription of ERE-CAT reporter gene, and an increase in tumor cell apoptosis and larger areas of tumor cell necrosis (Sartippour et al., 2006). Another study on the combined GTE and TAM reports a significant reduction in the number of hyperplastic alveolar nodules (HAN) and the number of argyrophilic nucleolar organization areas (AgNOR) (Sakata et al., 2011).

BD alone can inhibit the growth and infiltration of highly metastatic triple-negative human breast cancer cells *in vitro* and *in vivo* (Jiang et al., 2011; Jiang et al., 2012). In a study that adopted the MCF-7 cell xenograft model, it was reported that TAM and BD co-treatment could significantly enhance cell apoptosis, suppress tumor growth, and increase the expression of TAM resistance proteins BRAF and p21 (Cheng et al., 2017). Among all CAM products used by breast cancer patients, flaxseed (FS) is the third most commonly used (Boon et al., 2007). FS has two major anti-cancer components, secoisolariciresinol diglucoside (SDG) lignan and flaxseed oil (FO). When used in combination with TAM, it can effectively reduce the growth of MCF-7 breast tumors in ovariectomized (OVX) athymic mice at low circulating estrogen levels (Saggar et al., 2010). The underlying mechanism includes ER and growth factor-signaling pathways and involves proteins such as phosphorylated mitogen-activated protein kinase, PAKT, BCL2, and angiogenesis and vascular endothelial growth factor (Saggar et al., 2010).

#### 3.2.2 Increasing Sensitivity to TAM and Reducing Resistance

TAM is an endocrine drug that is most commonly used to block the effects of estrogen at all stages of breast cancer, especially in postmenopausal patients. While the ERα status has been used to identify breast cancer patients who may respond to TAM, there are still 30%–50% of treated Erα-positive breast cancer cells that show resistance to TAM (Early Breast Cancer Trialists' Collaborative Group, 1998). Therefore, in the experimental studies, many TAM-resistant cell lines were derived. At present, the mechanism of TAM resistance mainly includes the alteration or loss of ER expression; the activation or inactivation of various signal pathways involved in cellular processes, such as survival, proliferation, stress response, cell cycle, apoptosis inhibition regulated by the BCL-2 family, autophagy, and changes in mRNA expression (Rondón-Lagos et al., 2016).

A study involving the TAM-resistant cells named MCF-7^TR5^ (TAM resistant to 5 μM) reported that Z-ligustilide can enhance the inhibitory effect of TAM on the cell viability in MCF-7^TR5^ and sensitized MCF-7^TR5^ cells to TAM *via* promoting caspase-independent cell death (Qi et al., 2017). The study also reported that the combined use of Z-ligustilide with TAM can also induce DNA damage and apoptosis by restoring Nur77 expression and proteins degraded by autophagy in MCF-7^TR5^ (Qi et al., 2017).

Curcumin is a bioactive compound of the Chinese medicine turmeric that exhibits effective anti-cancer activity. Curcumin and its analogs also exhibit multi-target biological effects and have been used to enhance targeted therapy to assist breast cancer chemotherapy treatment (Nagaraju et al., 2012). A study involving the TAM-resistant cell lines named MCF-7/LCC2 and MCF-7/LCC9 reported that the combined use of curcumin and TAM resulted in synergistic growth inhibition, an increase in the sensitivity of MCF-7/LCC2 and MCF-7/LCC9 cells to TAM, and a reduction in IC_50_ of TAM (Jiang et al., 2013).

*Antrodia cinnamomea* (AC) is a medical mushroom that could have a novel role in breast cancer management (Lin et al., 2018). In a study involving TAM-resistant cell line MCF-7 tam-R, while 10^–6^ M TAM has no proliferation inhibitory effect on MCF-7 tam-R, the combined treatment of 10^–6^ M TAM with ethanol extract of AC was able to increase the effect of TAM to inhibit the proliferation of MCF-7 tam-R cells (Lin et al., 2018).

Evn-50 is an ethyl acetate extract of *Vitex negundo* L. When used in combination with TAM to treat TAM-resistant MCF-7/TAM-R cells, it significantly reduces cell viability, inhibits cell growth, induces apoptosis, and reverses TAM resistance of MCF-7/TAM-R cells (Hu et al., 2012). The study reports a decrease in phosphorylated AKT and p-MAPK44/42 levels, while total AKT and MAPK44/42 expressions were stable. This highlights that the underlying mechanism may be related to the downregulation of AKT and MAPK signaling pathways (Hu et al., 2012).

The combined use of persin and 4-OHT on TAM-R cells showed a synergistic induction of drug-resistant cell apoptosis pre-dominantly mediated by the CHOP-dependent endoplasmic reticulum stress (ERS) signaling cascades (McCloy et al., 2013). Jaeumkanghwa soup (JEKHT) is a compound composed of 12 traditional herbs (Jung et al., 2010). In antiestrogen-resistant LCC9 human breast cancer cells, the combination of JEKHT with 4-OHT can increase the sensitivity of the cells to TAM (De Oliveira Andrade et al., 2019). All the above evidence shows that natural or plant products can increase the sensitivity of drug-resistant cell lines to TAM.

On the contrary, some studies showed no significant interactions between TAM and certain natural products. In ovariectomized athymic mice with established MCF-7 that had only TAM implanted, increased biomechanical bone strength and bone mineral content (BMC) and density (BMD) in the femur and lumbar spine were reported (Chen et al., 2011b). In the mice group receiving a combination of SDG and FO diet with TAM therapy, no effects on the BMC and BMD of the femur or vertebrae were reported, indicating that the combined use of FS and TAM exhibited no obvious effect on bone health (Chen et al., 2011b).

Jiawei Xiaoyao San (JWXYS) is a very common Chinese herbal formula used by the Chinese for thousands of years (Chen et al., 2014). In MCF-7 cells, a study reports no statistically significant difference between TAM only and combined JWXYS with TAM groups in terms of cell number, cell cycle progression, and cell proliferation signal proteins such as AKT, ERK, P38, p27 (Kip1), and light chain (LC3) I, II (Chen et al., 2014). In the MCF-7 xenograft mouse model, the study found no significant change in tumor weight and the protein expression levels of AKT and ERK when compared to the TAM-only group, indicating no obvious interaction between JWXYS and TAM *in vivo* (Chen et al., 2014).

A study utilizing uterotrophic assay in 21-day immature female SD rats reports that the combined use of EGb 761 and TAM showed no significant effect on absolute or relative uterine weight, luminal epithelial cell height (LECH), and luminal circumference (LCO), indicating that EGB761 has no agonism nor antagonism on utero (Moon et al., 2018).

#### 3.2.3 Reducing the Side Effects of TAM

Although the use of TAM as an adjuvant endocrine therapy has effectively reduced the recurrence of ER+ breast patients and improved their survival rate, more and more studies have reported a series of side effects related to TAM that include hot flashes, night sweats, vaginal dryness, sleep, and emotional problems (Lorizio et al., 2012). Occasionally, more severe adverse events may occur, including endometrial cancer (Fisher et al., 1994; Kloos et al., 2002) and abnormal liver function (Yang et al., 2013). However, some natural or plant products can reduce the side effects of TAM, as shown in Table 5.

##### 3.2.3.1 Hot Flashes

A clinical observation study assessing the effect of *Actaea racemosa* L. [Ranunculaceae] (CR BNO 1055) and TAM combination on the number and intensity of hot flashes found that the number and severity of hot flashes decreased in patients after 12 months of combined use (Hernández Muñoz & Pluchino, 2003). Only 24.4% of the patients in the group receiving combined therapy reported severe hot flashes, while 73.9% of the patients in the control group reported severe hot flashes, showing that the combination of TAM and CR BNO 1055 for 12 months can provide a satisfactory reduction of the number and severity of hot flashes (Hernández Muñoz & Pluchino, 2003). A prospective observational study on the combined use of black cohosh (*Actaea racemosa* L.) and TAM in the treatment of breast cancer observed improvement in symptoms of hot flashes, sweating, sleep problems, and anxiety after 6 months of treatment; 90% of the study participants reported “very good/good” tolerability to black cohosh extract (Rostock et al., 2011).

##### 3.2.3.2 Endometrial Disease

In retrospective study of all breast cancer patients who received TAM treatment in the Taiwan Health Insurance Research Database between 1 January 1998 and 31 December 2008, it was found that among breast cancer survivors aged 20–79, the risk of endometrial cancer in patients treated with *Angelica sinensis* (Oliv.) Diels is lower when compared with patients who have never used *Angelica sinensis* (Oliv.) Diels (HR: 0.61, 95% CI: 0.44–0.84) (Wu et al., 2014). When compared with people who have never used Chinese herbal medicine, breast cancer survivors who take Chinese herbal medicines containing coumestrol, genistein, or daidzein concurrently with TAM therapy have no higher risk ratio for the subsequent development of endometrial cancer (Hu et al., 2015). More than half of the female breast cancer patients receiving TAM have used at least a Chinese herbal product (CHP). Two of the most commonly used CHPs are Jiawei Xiaoyao San and Shujing Huoxue Decoction (Tsai et al., 2014). Compared with non-CHP users, the risk ratio for the development of endometrial cancer among CHP users was 0.50 (95% CI = 0.38–0.64) (Tsai et al., 2014). The above results indicate that the combination of natural or plant products with TAM may reduce the risk of endometrial cancer after breast cancer.

JEKHT is a compound consisting of 12 traditional herbs (Jung et al., 2010). In a study adopting DMBA-induced ER+ breast cancer rat model, it was reported that the combined treatment of JEKHT with TAM can reduce risk of recurrence and developing TAM resistance, prevent the development of precancerous endometrial lesions, and prevent the immunosuppressive effects of TAM (De Oliveira Andrade et al., 2019). When compared with the TAM-only group, the combination of JEKHT and TAM significantly downregulated *Il-6*, *Foxp3*/T regulatory cell (Treg) markers, and *Tgfβ*1 that activate Treg (De Oliveira Andrade et al., 2019).

##### 3.2.3.3 Liver Damage

In TAM-intoxicated rats, a study found significantly increased levels of serum glutamic-oxaloacetic transaminase (sGOT), serum glutamic-pyruvic transaminase (sGPT), alkaline phosphatase (ALP), triglycerides (TG), cholesterol, urea, uric acid, bilirubin, and creatinine. The study proposed that the change in parameters may be related to extensive liver damage during TAM treatment (Rahate and Rajasekaran, 2015). *Desmostachya bipinnata* (L.) Stapf (PFDB) is used in Indian traditional medicine and is commonly called “sacrificial grass” in English (Rahate and Rajasekaran, 2015). The study reported that rats pre-treated with PFDB were effectively protected from TAM-induced hepatotoxicity. Reduced levels of the liver function test parameters, as discussed above, in serum were detected in these rats. Degree of liver cell necrosis was observed in these rats in histopathological studies (Rahate and Rajasekaran, 2015). The protective effect is dose-dependent (Rahate and Rajasekaran, 2015).

GTE also show hepatoprotective effect against TAM-induced liver injury in rats. A study where GTE was administered to TAM-intoxicated rats reported significant increase in antioxidant enzymes, reduction of glutathione concomitant, and significant decrease in the levels of thiobarbituric acid reactive substance (TBARS) and liver transaminase (El-Beshbishy, 2005a).

Rats treated with TAM showed elevated aminotransaminase, carbonyl groups (CGs), and 8-hydroxydeoxyguanosine (8OHdG) (Codoñer-Franch et al., 2013). When dried apple snack enriched with mandarin juice was used in combination with TAM, restoration of oxidative damage to protein and DNA in liver were observed with a significant reduction in plasma and liver markers of oxidative stress recorded, exhibiting a protective effect on the oxidative stress induced by TAM in rats (Codoñer-Franch et al., 2013).

Dimethyl-4.4'-dimethoxy-5,6,5',6'-dimethylenedioxybiphenyl-2.2'-dicarboxylate (DDB), a structural analog of Schizandrin B and Schizandrin C, is one of many compounds isolated from Chinese herbal medicine *Schisandra chinensis* (Turcz.) Baill. (Kim et al., 1999). Oxidative stress status of TAM-intoxicated liver injury in rats is determined by the change in levels of antioxidant enzymes (glutathione-S-transferase, glutathione peroxidase, and catalase), glutathione, TBARS, liver transaminase, sGPT, and sGOT (El-Beshbishy, 2005a). DDB is found to alleviate the oxidative stress status of TAM-intoxicated liver injury in rats, with the study reporting a significant increase in antioxidant enzymes, a significant decrease in the activity of sGPT and sGOT, reduction of glutathione concomitant, and a significant reduction of TBARS and liver transaminases (El-Beshbishy, 2005b).

## 4 Natural Products With Estrogen-Like Effects

There is growing evidence on the potential benefits of complementary and alternative medicine usage in treatment of cancer (Horneber et al., 2012). Prevalence rate of CAM usage among breast cancer survivor was reported to fall within 60-70%; with herbal preparations, vitamin and diet supplements being the more common CAM products used (Matthews et al., 2007).

While some natural products have a better effect on alleviating the adverse reactions during TAM treatment, there are some natural or plant products that have estrogen-like effects and mutual adverse effects when combined with TAM (Table 6).

### 4.1 Reduce Anti-Cancer Effect

*In vivo* Xanthorrhizol is a sesquiterpene compound isolated from turmeric and has been reported to have antimicrobial, antibacterial, anti-metastatic, and anti-inflammatory effects (Oon et al., 2015). In a study adopting TAM-treated MCF-7-implanted athymic nude mouse model, significantly increased tumor volume, larger tumor size, and increased protein expression of P38 and P27 (Kip1) were found in the group receiving combined treatment of Xanthorrhizol and TAM compared to the group receiving TAM only (Noomhorm et al., 2014)**.**

Si-Wu-Tang (SWT) is a Chinese herbal medicine composition consisting of *Angelica sinensis* (Oliv.) Diels, *Paeonia lactiflora* Pall., *Ligusticum striatum* DC., and *Rehmannia glutinosa* (Gaertn.) DC. (Chen et al., 2013). In a study using an MCF-7-implanted female athymic nude mouse model, it is found that the combination of SWT and TAM can reverse the anti-proliferative effect of TAM on tumor weight and tumor volume and increase the expression of estrogen receptor α and N-cadherin (Chen et al., 2013). The combination of SWT and TAM also increased protein expressions of ERK, AKT, P38, and p27 (Kip1), which could indicate the phytoestrogenic effect of SWT that could contribute to adverse effects when SWT interacts with TAM in the body (Chen et al., 2013)

Baihe Zhimu Tang (BZ) is a traditional Chinese medicine formula that consists of *Lilium brownii* var. *viridulum* Baker and *Anemarrhena asphodeloides* Bunge, often used to treat depression (Li et al., 2019). In the mouse xenograft model of human breast cancer MCF-7 cells, BZ reduced the effectiveness of TAM in the treatment of breast cancer and the concentration of endoxifen and 4-OH-tamoxifen in tumor-bearing mice (Li et al., 2019). The BZ formula and its major component, mangiferin, also significantly inhibited the activity of CYP450 enzymes in rat liver microsomes (Li et al., 2019).

In female nude mice inoculated subcutaneously with MCF-7/6 human breast adenocarcinoma cells, the combination of tangeretin and TAM counteracted the tumor growth inhibition effect of TAM (Bracke et al., 1999). This evidence reports adverse effects that occur between the interaction of certain herbal or plant products with TAM that doctors and patients should pay attention to during treatment of ER+ breast cancer.

## 5 Conclusion

This review has covered the potential interactions of TAM with 33 commonly used natural or plant products and 5 commonly used herbal formulas. With further in-depth research on breast cancer treatment drugs, much evidence reports that the combination of natural or plant products with conventional breast cancer treatment drug TAM contributes to the increased anti-tumor effect, inhibition of tumor cell proliferation, sensitizing breast cancer cells to TAM, and minimizing adverse effect of drugs. However, there are also studies that report no effect or adverse effects when certain natural or plant products are combined with TAM.

While there are significant discoveries and advancements in the study of drug combinations, there are still limitations in the experimental design of some studies. Firstly, while most *in vitro* studies adopted single cell line models, *in vitro* tumor environments tend to be vastly different, limiting the relevance of the research evidence. Secondly, studies on the mechanism of synergistic anti-tumor effect need to be further elucidated, particularly on contributions by different signaling pathways. A possible approach is to compare and analyze the structure-activity relationships of phytochemicals, TAM, and estradiol with chemical structures diagram retrieved from National Center for Biotechnology Information (2022). Table 7 includes some of the phytochemicals that have been investigated in the selected reviewed studies. Studies have shown that just the monitoring of protein expression levels is insufficient in understanding transcription activity *in vitro* (Gordân et al., 2009).

**TABLE 7:** Chemical structures of TAM, phytochemicals mentioned in the review.

TAM, plant species, plane products	Studies involved	Phytochemical, compounds, metabolites, and derivatives	IUPAC Name^a^	Chemical structure^a^
TAM	Klein et al. (2013)	Afimoxifene, 4-hydroxy-tamoxifen	4-[1-[4-[2-(Dimethylamino)ethoxy]phenyl]-2-phenylbut-1-enyl]phenol	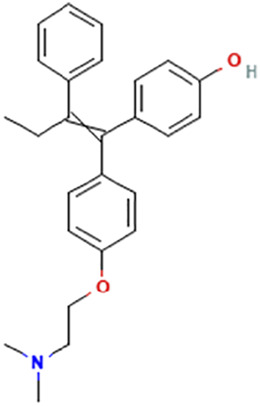
	Klein et al. (2013)	N-Desmethyl-tamoxifen	2-[4-[(Z)-1,2-Diphenylbut-1-enyl]phenoxy]-N-methylethanamine	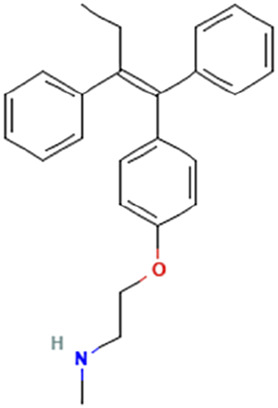
Sheng Ma (*Endosamara racemosa* (Roxb.) R. Geesink)	Shin et al. (2008)	Morin	2-(2,4-Dihydroxyphenyl)-3,5,7-trihydroxychromen-4-one	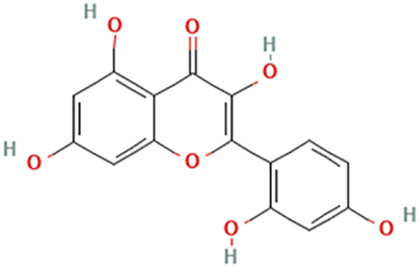
Green tea (*Camellia sinensis* (L.) Kuntze [Theaceae])	(El-Beshbishy, 2005a) (Sakata et al., 2011) (Sartippour et al., 2006) (Shin & Choi, 2009)	Epigallocatechin gallate (EGCG)	[(2R,3R)-5,7-Dihydroxy-2-(3,4,5-trihydroxyphenyl)-3,4-dihydro-2H-chromen-3-yl] 3,4,5-trihydroxybenzoate	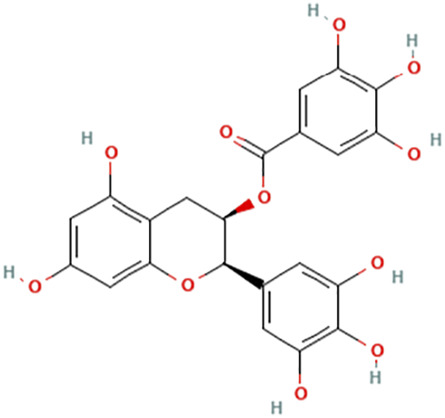
*Silybum marianum* (L.) Gaertn	Kim et al. (2010)	Silibinin/silybin	(2R,3R)-3,5,7-Trihydroxy-2-[(2R,3R)-3-(4-hydroxy-3-methoxyphenyl)-2-(hydroxymethyl)-2,3-dihydro-1,4-benzodioxin-6-yl]-2,3-dihydrochromen-4-one	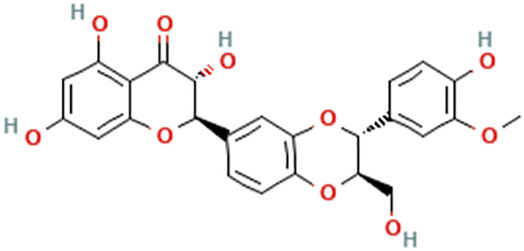
*Myrica rubra *(Lour.) Siebold & Zucc	Li et al. (2011a)	Myricetin	3,5,7-Trihydroxy-2-(3,4,5-trihydroxyphenyl)chromen-4-one	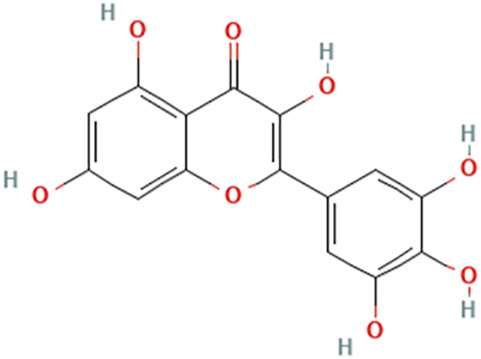
Huang Qin (*Scutellaria alpina* L.)	Li et al. (2011b)	Baicalein	5,6,7-Trihydroxy-2-phenylchromen-4-one	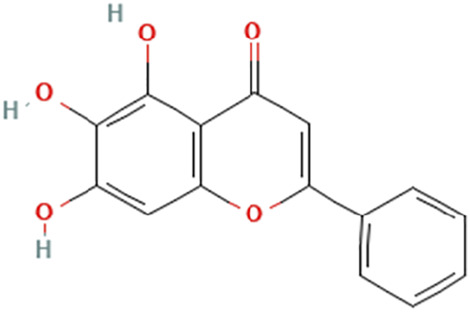
Turmeric (*Curcuma longa* L.)	(Cho et al., 2012) (Jiang et al., 2013) (Nagaraju et al., 2012)	Curcumin	(1E,6E)-1,7-bis(4-Hydroxy-3-methoxyphenyl)hepta-1,6-diene-3,5-dione	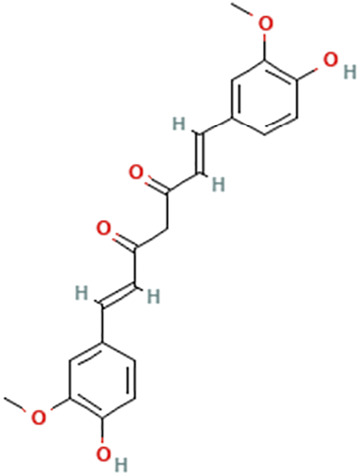
	(Noomhorm et al., 2014) (Oon et al., 2015)	Xanthorrhizol	2-Methyl-5-[(2R)-6-methylhept-5-en-2-yl]phenol	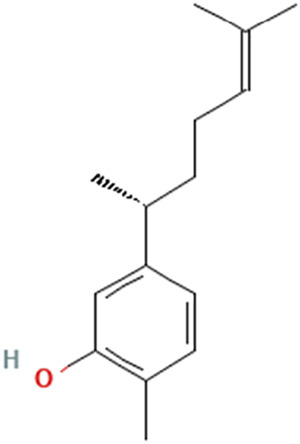
Study involving Kaempferol did not specify plant species in which Kaempferol is derived from. ^a^The flavonoid may be found in *Panax ginseng *C.A.Mey. and *Epimedium perralderianum* Coss	Piao et al. (2008)	Kaempferol	3,5,7-Trihydroxy-2-(4-hydroxyphenyl)chromen-4-one	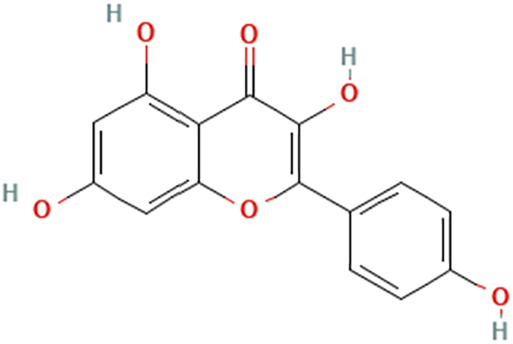
Quercetin	Shin et al. (2006)	Quercetin	2-(3,4-Dihydroxyphenyl)-3,5,7-trihydroxychromen-4-one	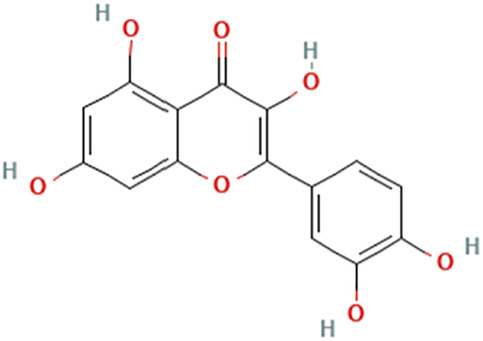
*Ginkgo biloba*	(Dias et al., 2013) (Jacobs and Browner, 2000) (Moon et al., 2018) (Vardy et al., 2013)	Studies did not specify a particular compound	Studies did not specify a particular compound	Studies did not specify a particular compound
*Fucus vesiculosus* Linnaeus, *Cladosiphon okamuranus Tokida*, *Laminaria saccharina* f. linearis J.Agardh	(Atashrazm et al., 2015) (Zhang et al., 2013)	Fucoidan	[(2S,3S,4S,5S,6R)-4-Hydroxy-5-methoxy-2,6-dimethyloxan-3-yl] hydrogen sulfate	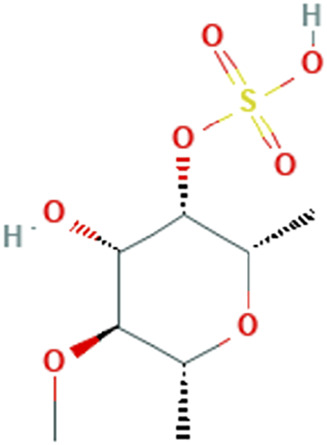
Mistletoe (*Viscum album* L.)	(Kienle et al., 2009) (Tröger et al., 2014) (Weissenstein et al., 2019)	Studies used extracts of *Viscum album* L. (VAE)	Studies used extracts of *Viscum album* L. (VAE) that contains a variety of phytochemicals	Study by Kienle et al., 2009 has broken down some VAE compounds in
Soy, soya studies did not specify a species, referring to soy in general	(Tanos et al., 2002) (Mai et al., 2007)	Isoflavone genistein	5,7-Dihydroxy-3-(4-hydroxyphenyl)chromen-4-one	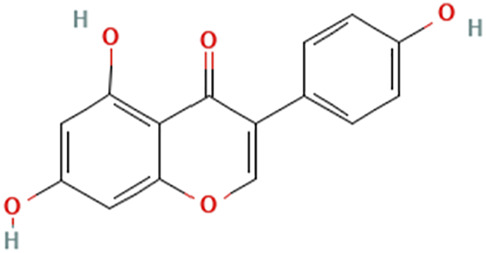
Avocado (*Persea americana* Mill.)	Roberts et al. (2007)	Persin	[(12Z,15Z)-2-Hydroxy-4-oxohenicosa-12,15-dienyl] acetate	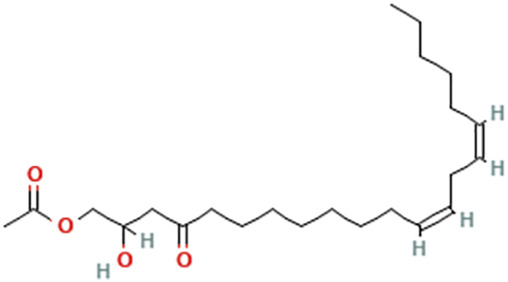
Canola oil (*Brassica napus* L.)	Cho et al. (2010)	Alpha-linolenic acid	(9Z,12Z,15Z)-Octadeca-9,12,15-trienoic acid	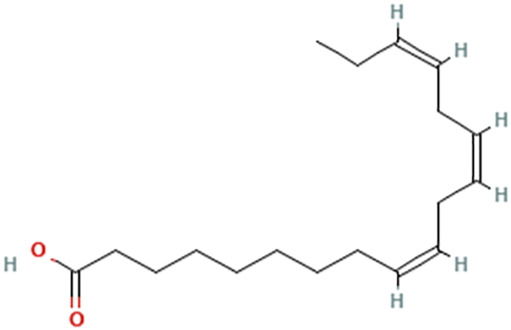
		Oleic acid	(Z)-Octadec-9-enoic acid	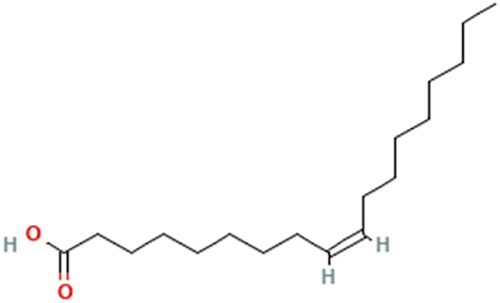
		Omega-3	(4Z,7Z,10Z,13Z,16Z,19Z)-Docosa-4,7,10,13,16,19-hexaenoic acid; (5Z,8Z,11Z,14Z,17Z)-icosa-5,8,11,14,17-pentaenoic acid; (9Z,12Z,15Z)-octadeca-9,12,15-trienoic acid	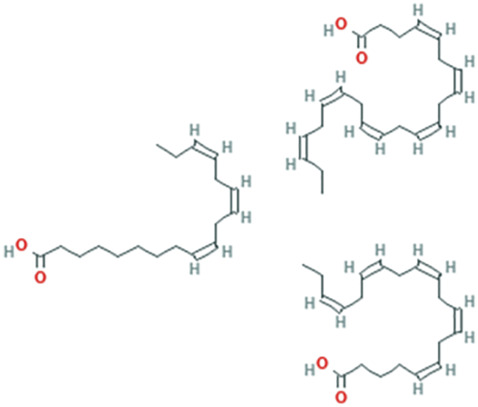
		Omega-6	(5Z,8Z,11Z,14Z)-Icosa-5.8,11,14-tetraenoic acid; (9Z,12Z)-octadeca-9,12-dienoic acid	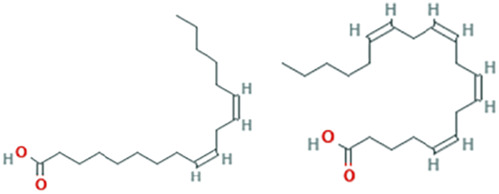
*Angelica sinensis* (Oliv.) Diels [Apiaceae]	Ma et al. (2017)	Z-Ligustilide	(3Z)-3-Butylidene-4,5-dihydro-2-benzofuran-1-one	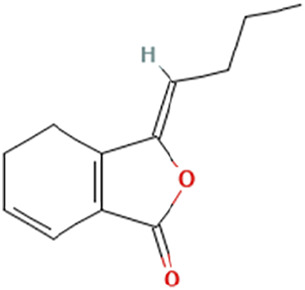
*Strobilanthes crispus* (*Strobilanthes crispa* (L.) Blume)	(Yaacob et al., 2014) (Yaacob et al., 2015)	Study looks into the effects of a subfraction of *Strobilanthes crispus* leaves	Multiple compounds in bioactive subfraction, Have not been conclusively determined in study	Multiple compounds in bioactive subfraction. Have not been conclusively determined in study
*Antrodia cinnamomea*	Lin et al. (2018)	Antrodin C	1-Hydroxy-3-[4-(3-methylbut-2-enoxy)phenyl]-4-(2-methylpropyl)pyrrole-2,5-dione	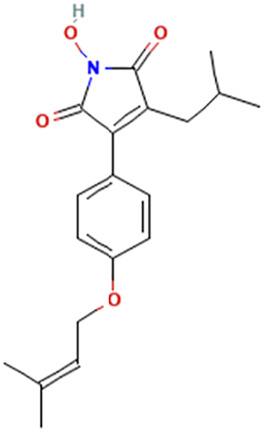
		Antcin B	2-Methyl-3-methylidene-6-[(4S)-4,10,13-trimethyl-3,7,11-trioxo-1,2,4,5,6,12,14,15,16,17-decahydrocyclopenta [a]phenanthren-17-yl]heptanoic acid	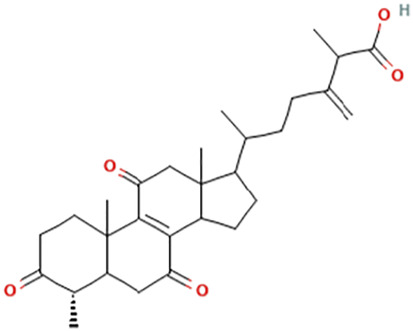
		Antcin C	(6R)-6-[(4S,5S,7S,10S,13R,14R,17R)-7-Hydroxy-4,10,13-trimethyl-3,11-dioxo-2,4,5,6,7,12,14,15,16,17-decahydro-1H-cyclopenta [a]phenanthren-17-yl]-2-methyl-3-methylideneheptanoic acid	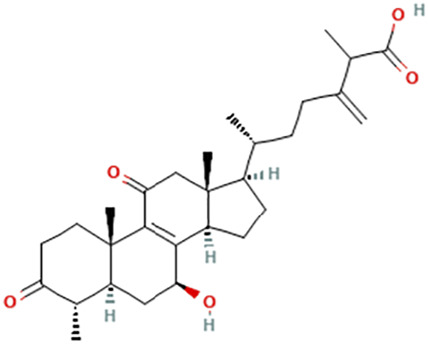
		Antcin K	(6R)-2-Methyl-3-methylidene-6-[(3R,4R,5R,7S,10S,13R,14R,17R)-3,4,7-trihydroxy-4,10,13-trimethyl-11-oxo-2,3,5,6,7,12,14,15,16,17-decahydro-1H-cyclopenta [a]phenanthren-17-yl]heptanoic acid	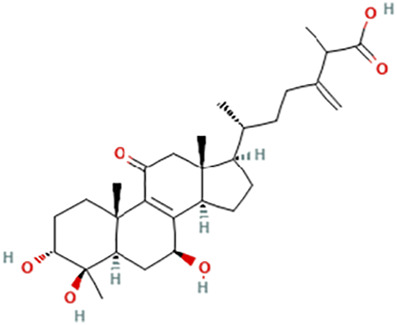
		Methyl antcinate B	Methyl (6R)-2-methyl-3-methylidene-6-[(4S,10S,13R,14R,17R)-4,10,13-trimethyl-3,7,11-trioxo-1,2,4,5,6,12,14,15,16,17-decahydrocyclopenta [a]phenanthren-17-yl]heptanoate	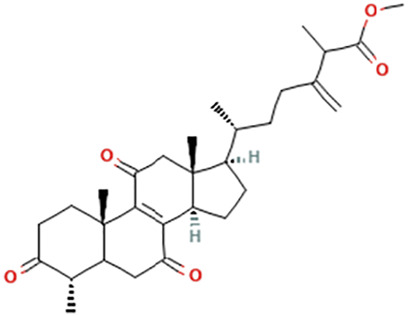
		Eburicoic acid	(2R)-2-[(3S,5R,10S,13R,14R,17R)-3-Hydroxy-4,4,10,13,14-pentamethyl-2,3,5,6,7,11,12,15,16,17-decahydro-1H-cyclopenta [a]phenanthren-17-yl]-6-methyl-5-methylideneheptanoic acid	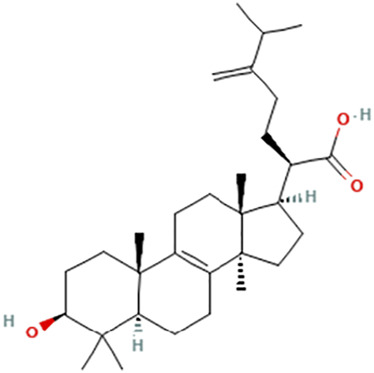
		Dehydroeburicoic acid	(2R)-2-[(3S,5R,10S,13R,14R,17R)-3-Hydroxy-4,4,10,13,14-pentamethyl-2,3,5,6,12.15,16,17-octahydro-1H-cyclopenta [a]phenanthren-17-yl]-6-methyl-5-methylideneheptanoic acid	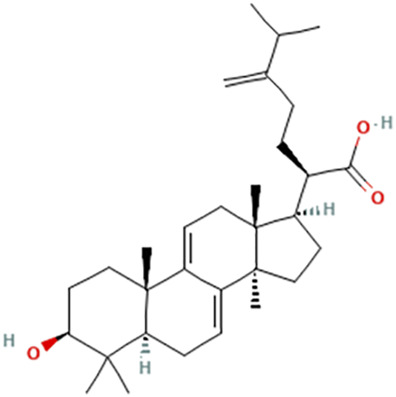
Huaier (*Trametes robiniophila* Murr.)	(Pan et al., 2019) (Qi et al., 2016)	Pan et al. (2019) highlighted that other than proteoglycan, detailed effective bioactive components remain elusive	Pan et al. (2019) tentatively named the compound proteoglycan TPG-1	The compound has a molecular mass of ∼5.59 × 10^4^ Da; 43.9% and 41.2% total carbohydrate and protein compositions, respectively
Red clover (*Trifolium pratense* L.)	(Khazaei & Pazhouhi, 2019) (Vlaisavljevic et al., 2014)	The study did not specify a certain phytochemical it is looking into. Study used *T. pratense* extract. Khazaei & Pazhouhi (2019) documented the chemical profile of the extract using high-performance liquid chromatography–ultraviolet (HPLC-UV) chromatogram	-	Results of the chromatogram show that coumarins, pterocarpans, flavonoids, isoflavones, coumarins, and tyramine are present in the *T. pratense* extract. Various isoflavones have been identified in the extract, including afrormosin, biohanin A, daidzein, calycosin, formononetin, genistein, irilin B, irilone, pratensein, prunetin, pseudobaptigenin, methylorobol, and texasin
*Fructus schizandra*	(El-Beshbishy, 2005b) (Kim et al., 1999)	Schizandrin B	3.4,5,19-Tetramethoxy-9,10-dimethyl-15,17-dioxatetracyclo[10.7.0.0^2,7^.0^14,18^]nonadeca-1 (19),2,4,6,12.14 (18)-hexaene	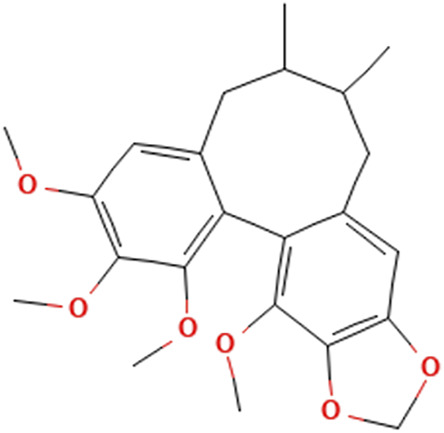
		Schizandrin C	(12*S*,13*R*)-3,22-Dimethoxy-12,13-dimethyl-5.7,18,20-tetraoxapentacyclo [13.7.0.0^2,10^.0^4,8^.0^17,21^]docosa-1(22),2.4(8),9,15,17 (21)-hexaene	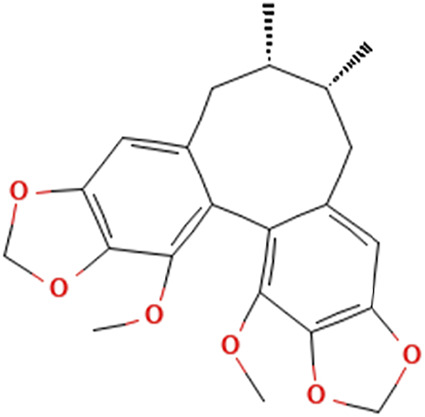

a IUPAC, name, and chemical structures are retrieved from National Center for Biotechnology Information (2022).

Additionally, there are still issues relating to herbal formulations and their composition that is often unaddressed, such as the clarification of herbal formula composition and quality control of herbal products. Moreover, in studies that investigate herbal formulations or plant extracts, there is increased complexity in the determination of which combinations of compounds are producing the observed additive, synergistic, or antagonistic effects.

Natural or plant products have been used in clinical practice for thousands of years, with numerous modern-day studies reporting the anti-cancer effects of such herbal medicines and compounds. While continuous efforts in scientific and laboratory investigations of individual herbal and herbal compositions in breast cancer treatment have given rise to various evidence from the derivation of major anti-cancer components and especially evaluation of effects from combination with conventional breast cancer treatment drugs, it is necessary to conduct further research and review them systematically, in particular, clinical studies to verify the herb–drug interactions. In this regard, cooperation between medical doctors, traditional Chinese medicine practitioners, and allied health practitioners that use herbal products to monitor and evaluate the use of natural or plant products in patients receiving TAM endocrine treatment for breast cancer is important to ensure the effectiveness of the treatments and the safety of patients. Such efforts, including further research, are critical for the integration of the scientific evidence into clinical practice to guide the use of natural or plant products and anti-cancer drugs such as TAM for better care of breast cancer patients.
